# From juniors to seniors: changes in training characteristics and aerobic power in 17 world-class cross-country skiers

**DOI:** 10.3389/fphys.2023.1288606

**Published:** 2023-11-20

**Authors:** Jacob Walther, Thomas Haugen, Guro Strøm Solli, Espen Tønnessen, Øyvind Sandbakk

**Affiliations:** ^1^ Department of Neuromedicine and Movement Science, Centre for Elite Sports Research, Norwegian University of Science and Technology, Trondheim, Norway; ^2^ Norwegian Ski Federation, Oslo, Norway; ^3^ Kristiania University College, Oslo, Norway; ^4^ Department of Sport Science and Physical Education, Nord University, Bodø, Norway; ^5^ School of Sport Science, UiT The Artic University of Norway, Tromsø, Norway

**Keywords:** long-term development, training characteristics, aerobic power, endurance sports, sex differences, training intensity distribution, periodization

## Abstract

**Purpose:** To compare training characteristics and aerobic power (VO_2max_) between the most successful junior and senior seasons of world-class cross-country (XC) skiers and to identify differences between sexes and among sprint and distance skiers.

**Methods:** Retrospective analysis was conducted on self-reported training and VO_2max_ tests of ten male and seven female world-class XC-skiers, collectively holding 38 Olympic medals. Training was categorized by form (endurance, strength, speed, other) and mode (specific, unspecific) and was divided into low- (LIT), moderate- (MIT), and high-intensity training (HIT).

**Results:** Total training increased by 203 ± 130 h (35% ± 31%, *p* < .001, large effect) and 78 ± 69 sessions (21% ± 24%, *p* < .001, very large effect). Junior training volume (658 ± 107 h) did not correlate with senior volume (861 ± 74 h) but correlated negatively with changes in volume (*r* = −.822, *p* < .001). No sex differences were observed related to total volume, but distance skiers increased their total volume more than sprint skiers (*p* = .037, large effect). Endurance training increased by 197 ± 117 h (*p* < .001; large effect) tied to increased low-intensity training (186 ± 115 h, *p* < .001; large effect) and moderate-intensity training (13 ± 7 h, *p* < .001; large effect). Training intensity distribution (% LIT/MIT/HIT) was 91/3/6 in junior and 92/4/4 in senior season. Women demonstrated greater increase of unspecific modes (100 ± 58 vs. 37 ± 44 h, *p* = .022; large effect) and strength training (25 ± 23 vs. −3 ± 17 h, *p* = .010, large effect). Men improved absolute (8% ± 5%; *p* = .009; large effect) and relative VO_2max_ (6% ± 4%; *p* = .016; large effect) from junior to senior, while women only increased relative VO_2max_ (7% ± 5%, *p* = .012; large effect).

**Conclusion:** This study provides novel information regarding changes in training characteristics and aerobic power from junior to senior age in world-class XC-skiers. Overall, the enhanced training volume during this transition was primarily driven by increased LIT and MIT and the exceptionally high relative VO_2max_ at junior age further increased in both sexes.

## Introduction

Endurance performance is mainly determined by aerobic power (VO_2max_), fractional utilization, and working economy (Joyner and Coyle, 2008). World-leading athletes have maximized these determinants through years with high volumes of systematic training. A wealth of research has illuminated the physiology (Sandbakk et al., 2016; Bell et al., 2017; Jones et al., 2021a) and training characteristics (Tønnessen et al., 2014; Erp et al., 2020; Haugen et al., 2022) of elite endurance athletes during their years of peak performance which is typically reached in the mid-to-late 20s (Allen and Hopkins, 2015; Haugen et al., 2018a; Walther et al., 2022). However, notably less information is available regarding the development of these factors from junior stages to peak performance, underscoring that existing talent development practices in endurance sports are built upon limited scientific evidence (Staff et al., 2023).

Cross-country (XC) skiing is an endurance sport where some of the highest VO_2max_values and training volumes are reported. Typical relative VO_2max_ values for world-class males and females are in the range of 80–85 and 65–70 mL kg^−1^·min^−1^, respectively (Tønnessen et al., 2015; Sandbakk et al., 2016). Senior world-class XC-skiers devote approximately 750–950 h per year to training (Tønnessen et al., 2014; Sandbakk and Holmberg, 2017; Solli et al., 2017), where ∼90% is endurance training and the remaining ∼10% is strength and speed training. The endurance training is commonly reported to include nearly 90% low-intensity training (LIT), 4%–5% moderate-intensity training (MIT) and 5%–6% high-intensity training (HIT). As the main competition approaches, there is a shift towards reduced volume and an augmented proportion of competition-specific intensity (Tønnessen et al., 2014; Solli et al., 2017). Around 50%–60% of endurance training is conducted in specific exercise modes during the general preparation, while this proportion escalates to 80%–90% during the competition phase (Sandbakk and Holmberg, 2017; Solli et al., 2017). Sprint-skiing specialists show a slightly different training pattern, as they execute slightly less training on an annual basis, less training in long uphills, and more focus on strength and speed training (Losnegard and Hallén, 2014).

Considerably less information is available regarding the training and physiological capacities of well-performing XC-skiers at younger age stages and potential sex differences in the development of both physiology and training. Relative VO_2max_ values in the range 65–70 and 55–60 mL kg^−1^·min^−1^ have been documented in 17 to 18-year-old male and female XC skiers, respectively (Sandbakk et al., 2011; Zoppirolli et al., 2020; Sollie and Losnegard, 2022). An annual endurance training volume of ∼600 and 4–500 h have been reported in well-trained 20- and 17-year-old junior skiers, respectively (Sandbakk et al., 2011; Karlsson et al., 2021). Furthermore, Seiler and Kjerland (Seiler and Kjerland, 2006) provided insights into the training intensity distribution (TID) of well-trained junior XC-skiers, revealing that approximately 75% of the endurance sessions were dedicated to LIT, 8% to MIT and 17% to HIT. Despite these foundational analyses, comprehensive insights into the training and performance determinants of junior skiers who ultimately ascend to world-class senior performance remain elusive. In essence, an immediate necessity exists for additional research centered on the development of endurance performance and associated physiological and training characteristics (Staff et al., 2023). Hence, this study seeks to retrospectively compare VO_2max_ and training characteristics among world-class XC-skiers in their trajectory from junior to senior age. Additionally, the study aims to unveil potential differences in training characteristics and the trajectory of VO_2max_ development across male and female skiers, as well as between sprint and distance specialists during the same time-period. Such information would substantially contribute to the refinement of talent development programs in endurance sports. Our main hypothesis is that the increase in training volume from junior to senior is primarily due to more LIT, while the corresponding increase in aerobic power is less pronounced.

## Materials and methods

### Participants

Seventeen senior XC-skiers (ten males and seven females) classified as world-class (Tier 5) according to performance caliber classification by McKay et al. (McKay et al., 2022) were recruited from the researchers’ network. Inclusion criteria were as follows: 1) medalist in either world championships or Olympic games, or repeatedly on the podium in world-cup races, 2) detailed training diary data of their most successful junior and senior seasons. The skiers included in this study were born between 1972 and 1998 and had won 38 Olympic medals, 69 senior world championship titles, 291 world cup victories and 23 junior world championship titles. In total 16 of the participants had participated in the junior world championships and 15 achieved at least one medal. Of the 17 skiers, 12 (six males and six females) where distance specialists and five (four males and one female) were sprint specialists. The mean age was 19.9 ± 0.3 years in the analyzed junior season and 28.4 ± 2.7 years in the senior season. The average International Ski Federation (FIS) points of the three best races combining sprint and distance races were 44.0 ± 18.3 during the junior season and 6.34 ± 9.3 during the senior season (International Ski Federation, 2023). All participants provided their written informed consent to participate in this study. The Regional Committee for Medical and Health Research Ethics waived the requirement for ethical approval for this study. The ethics of the project was performed according to the institutional requirements at the Department of Neuromedicine and Movement Science, Norwegian University of Science and Technology, Norway. Approval for data security and handling was obtained from the Norwegian Centre for Research Data (reference number 419807).

### Study design and selection of seasons

A retrospective study design was used to compare self-reported training characteristics and aerobic power between the most successful junior and senior season. The most successful senior season of each athlete was selected holistically taking several performance parameters into account. The parameters were assessed in the following order with available training data assumed: 1) number of individual medals in world championships and Olympic Games, 2) number of world cup podiums, 3) number of team medals in world championships and Olympic Games, and 4) calculated peak age. For the selection of junior season, the following order was applied: 1) number of individual medals in junior world championships, 2) number of team medals in junior world championships, 3) number of podium spots in national junior races. Age was determined in accordance with FIS competition rules, while peak age was calculated based on individual FIS point trajectories according to (Walther et al., 2022). FIS point analyses were conducted as described previously (Walther et al., 2022). Due to the introduction of FIS points that occurred after they turned senior, two skiers were excluded from the FIS point analyses.

### Training data

**FIGURE 1:**
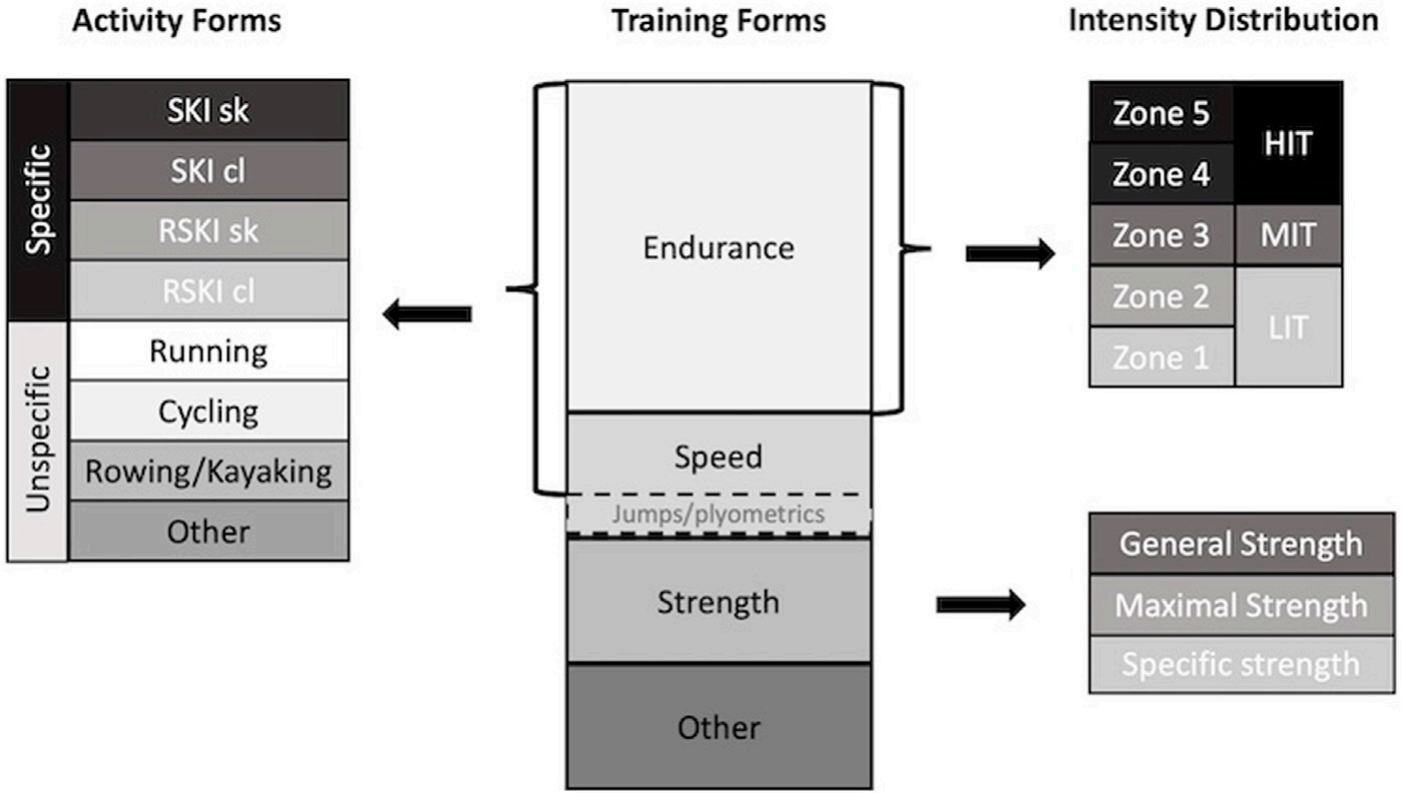
Distribution methods of total training across training forms, modes, and intensities modified from (Tønnessen et al., 2014). Other training forms refers to all registered training not related to endurance training (e.g., mobility or football). Skiing (SKI) and roller-skiing (RSKI) performed in skating (sk) and classic (cL) style were classified as specific, running, cycling, rowing as unspecific modes. Other unspecific activity forms refer to all other registered training related to endurance training (e.g., swimming).

All participants reported their training in a web-based diary designed by the Norwegian Olympic Federation (Olympiatoppen). Prior to the introduction of this diary the older participants had used a non-web-based version (Microsoft Excel) or written diaries designed by the Norwegian Ski Federation (NSF) including the same categories for registration. The recorded training included information about frequency, duration, training form (endurance, strength, speed, and other [e.g., mobility or football]), intensity, and the exercise modes [specific (skiing and roller-skiing) and unspecific (running, cycling, kayak, rowing, and other including all other endurance training forms like, e.g., swimming)] for the endurance and speed training. The distribution of training was adapted from a previous study (Tønnessen et al., 2014) as presented in Figure 1. Intensity and duration of endurance training was registered by using the modified session goal approach (Sylta et al., 2014b) and in accordance with the five-zone-model outlined by Olympiatoppen. Maximal heart rate was determined based on a combination of physical tests and the highest heart rate achieved in high intensity sessions or competitions within the junior and senior season investigated. These methods have previously been shown to provide valid and accurate measurements of endurance training in XC-skiers (Sylta et al., 2014a). However, as the intensity zone boundaries are not clearly anchored in underlying physiological events, data were analyzed using a three-zone-scale where LIT included zone 1 and 2, MIT included zone 3 and HIT included zone 4 and 5. Passive recovery during intervals was not recorded whereas active recovery was recorded as LIT. As for the endurance training, the speed and strength training were also registered time-based. For speed training within endurance sessions, 2 minutes per bout were registered. For strength training, total duration of the session (including recovery periods) was recorded. All recorded training was digitized and cross-checked for correct registration and distribution. Consistency in reporting of training and correct application of the five-zone-model were verified via a semi-structured interview with each participant during the data-analysis phase of this study. To account for possible differences in periodization between junior and senior age, monthly data was analyzed instead of previously applied division into periodization phases. Due to incomplete day-to-day information during junior age, two of the skiers were not included in the examination of total number of sessions resulting in a final sample size of *n* = 15 for these analyses.

### Physiological testing

The participants underwent physiological testing regularly. All tests were conducted at Olympiatoppen in Oslo. Each test consisted of the same standardized VO_2max_ ramp test with testing procedures being similar at junior and senior age. Procedures and apparatus are described in detail elsewhere (Ingjer, 1991; Tønnessen et al., 2014). Criteria for VO_2max_ achievement were that at least three out of the four following conditions were achieved: a plateau or leveling off in VO_2_ despite increased exercise intensity, peak respiratory exchange ratio (RER) ≥ 1.05, blood lactate concentration (BLa) ≥ 8 mmol L^−1^, and rating of perceived exertion (RPE) of 19–20, in line with well-established procedures (Midgley et al., 2007; Edvardsen et al., 2014; Tønnessen et al., 2015). Body mass was measured before each test (Seca model 770; Seca, Hamburg, Germany). For comparisons of junior and senior age, the test with the highest VO_2max_ at junior age and the most successful season was selected. The phases in which the tests with the highest VO_2max_ occurred were not consistent across skiers (junior: 2 tests in the early and 11 in the late preparation phase; senior: 5 tests in the early preparation, 5 in the late preparation and 3 in the competition phase). However, as previously shown by Losnegard et al. (Losnegard et al., 2013) VO_2max_ remains relatively stable during a season among high-level skiers.

### Statistical analyses

All data is presented as mean ± standard deviation (SD). For better comparability, monthly training data was divided by the number of days in that month and multiplicated by 30.4. Normality of the data was tested by visual inspection of histograms and Shapiro-Wilk test (α = 0.05). Statistical comparisons between junior and senior season were assessed using paired sample *t*-test or its nonparametric counterpart the Wilcoxon-test. For comparisons between the ten males and seven females and between the five sprint and twelve distance skiers, unpaired *t*-test or its nonparametric counterpart, the Mann-Whitney-U test, were applied. The *p*-value was set to <.05. Pearson’s product coefficient was applied for correlations and effect sizes (ESs) of nonparametric tests. Correlation coefficients were interpreted according to (Hopkins et al., 2009) as follows: *r* < 0.1 = trivial, 0.1 to 0.3 = small, 0.3 to 0.5 = moderate, 0.5 to 0.7 = large, 0.7 to 0.9 = very large and >0.9 = extremely large. ESs for parametric tests were calculated as Cohen *d*, and criteria for interpretation were as follows: 0.0 to 0.2 trivial, 0.2 to 0.6 small, 0.6 to 1.2 moderate, 1.2 to 2.0 large and >2.0 very large (Hopkins et al., 2009).

## Results

### Total volume

**FIGURE 2:**
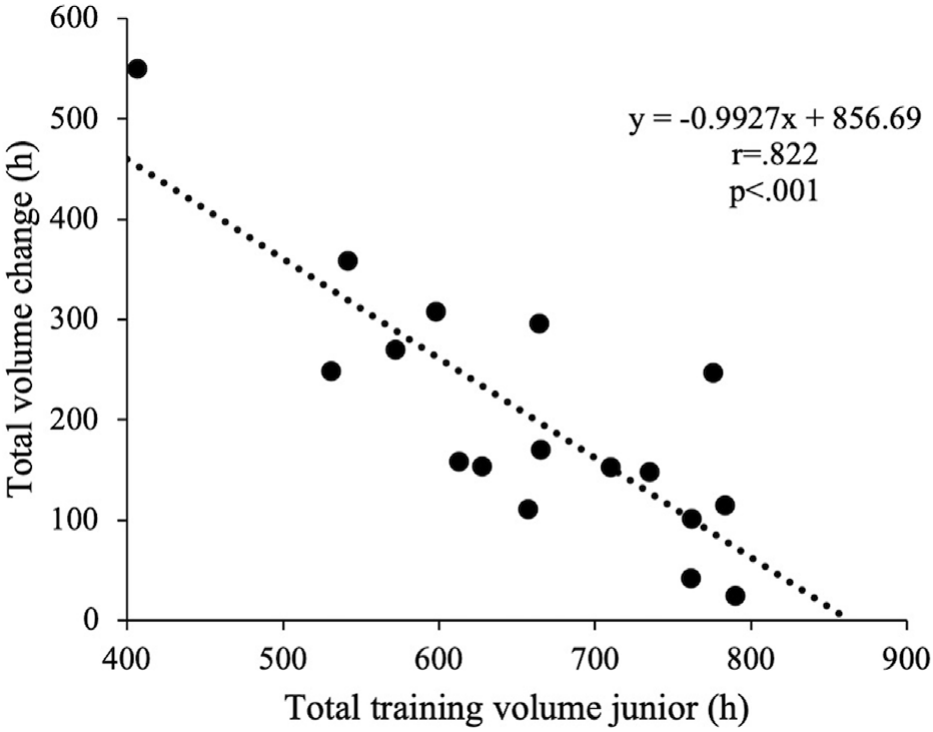
The relationship between total training volume at junior age and change in total training volume from junior to senior age in 17 world-class XC-skiers.

In total, 25.837 h of training were analyzed. Total training volume for the junior vs. the senior season was 658 ± 107 and 861 ± 74 h, corresponding to a mean increase of 203 ± 130 h (35% ± 31%, *d* = 1.6, *p* < .001) over an average time period of 8.5 ± 2.8 years. The training volume in junior season did not correlate significantly with training volume in senior season (*r* = .010, *p* = .969) but correlated negatively with change in training volume (Figure 2). The total number of logged sessions was 441 ± 71 during junior and 519 ± 34 during senior season corresponding to a change of 78 ± 69 sessions (21% ± 24%, *r* = 0.9, *p* < .001).

**FIGURE 3:**
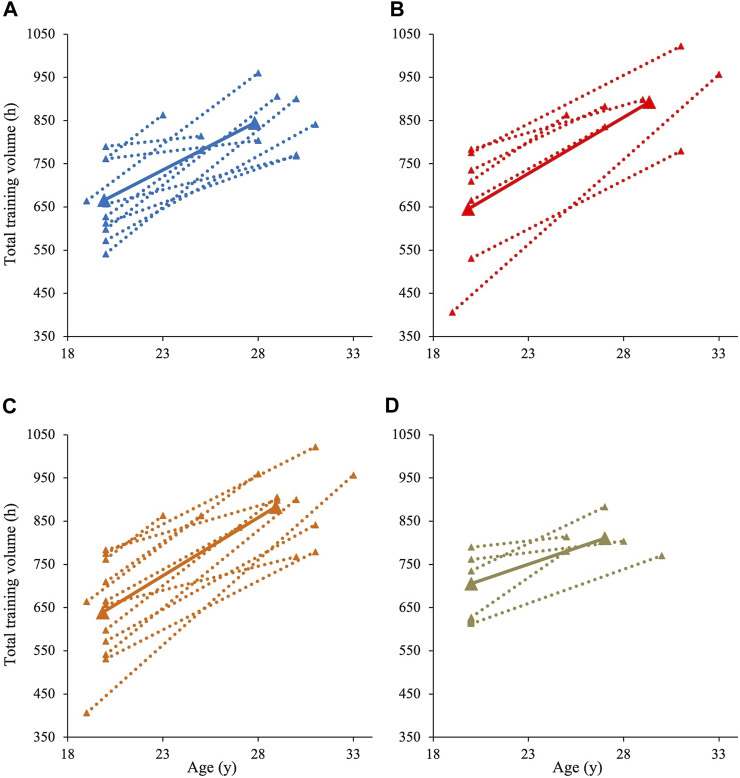
Individual (dotted line) and mean (solid line) change in total training volume in world-class XC-skiers from their best junior to their best senior season in men **(A)**, women **(B)**, distance skiers **(C)**, and sprint skiers **(D)**.

Figure 3 shows individual changes in total volume from junior to senior age. No significant differences were observed in total training volume between men and women neither in junior (658 ± 86 vs. 658 ± 140 h, *d* < 0.1) nor senior season (841 ± 65 vs. 891 ± 80 h, *d* = 0.7). Moreover, the change in total volume from junior to senior did not differ significantly between men and women (182 ± 118 vs. 233 ± 149 h, *r* = 0.1). Differences in total volume between distance and sprint skiers were not significant neither during junior (639 ± 114 vs. 705 ± 80 h, *d* = 0.6) nor senior season (883 ± 74 vs. 810 ± 44 h, *d* = 1.1). However, distance skiers showed a significantly larger increase in total volume than sprint skiers (244 ± 129 vs. 105 ± 66 h, *r* = 0.5, *p* = .037).

### Training forms

Table 1 shows the distribution of training forms at junior and senior age. The total volume of endurance training was 197 ± 117 h higher at senior age, with no significant sex differences neither at junior or senior age, nor in the changes The total volume of strength training did not differ between men and women at junior age. However, women increased total volume of strength training more than men (25 ± 23 vs. −3 ± 17 h, *d* = 1.5, *p* = .010) and performed more strength training at senior age (75 ± 19 vs. 41 ± 11 h, *r* = 0.7, *p* = .003). The observed sex differences in strength training during the senior season were significant for all months from June to January with highest differences during the middle preparation period (∼5 h in August). No sex differences were observed in speed and other training forms at neither junior age, senior age, or change. Sprint and distance skiers did not differ in any training form at any age, and the changes in these variables were not significant.

**TABLE 1:** Annual training distribution (mean ± SD) of training forms, modes, and intensities in world-class XC-skiers during most successful junior and senior season.

	Junior	Senior	*p*	Effect size *d*	Effect size *r*
Training forms					
Endurance (h)	584.4 ± 98.1	781.8 ± 62.0	<.001	1.7	
% of total training	88,7 ± 5.4	90.8 ± 2.7	.017	0.6	
Strength (h)	47.0 ± 15.6	55.3 ± 22.3	.174	0.3	
% of total training	7.1 ± 1.9	6.3 ± 2.3	.435		−0.2
Speed (h)	15.4 ± 7.9	18.5 ± 7.7	.188	0.3	
% of total training	2.4 ± 1.2	2.1 ± 0.9	.556	−0.1	
Other (h)	11.4 ± 6.5	5.5 ± 5.0	.003	−0.9	
% of total training	1.8 ± 1.1	0.7 ± 0.6	<.001	−1.1	
Exercise modes					
Specific (h)	406.2 ± 78.8	544.5 ± 66.8	<.001		0.9
Unspecific (h)	189.7 ± 37.1	252.8 ± 53.0	<.001	1.1	
% SPE/UNSPE	68/32	68/32			
Intensity distribution					
LIT (h)	530.2 ± 92.7	715.7 ± 59.0	<.001	1.6	
MIT (h)	20.3 ± 7.3	33.7 ± 8.6	<.001	2.0	
HIT (h)	34.0 ± 8.9	32.5 ± 6.7	.477	−0.2	
LIT/MIT/HIT (%)	91/3/6	92/4/4			

SPE, specific modes; UNSPE, unspecific modes; LIT, low intensity training; MIT, moderate intensity training; HIT, high intensity training.

### Endurance training

Information related to annual and monthly TID at junior and senior age is presented in Table 1 and Figure 5, respectively. The volume of both LIT (186 ± 115 h) and MIT (13 ± 7 h) increased from junior to senior, while no change in HIT was observed. No sex differences in TID were observed at junior or senior, nor in the change from junior to senior age. Mean volume of LIT, MIT and HIT at junior and senior age did not differ between sprint and distance skiers.

Figure 6 shows monthly distribution of applied exercise modes during junior and senior age. Specific training volume was 138 ± 100 h higher at senior age, while unspecific training increased with 63 ± 58 h (*d* = 1.1, *p* < .001) from junior to senior. No sex differences were observed in specific modes. However, women showed a larger increase (100 ± 58 vs. 37 ± 44 h, *d* = 1.3, *p* = .022) and a higher volume of unspecific modes at senior age (286 ± 60 vs. 230 ± 33 h, *d* = 1.2, *p* = .026). Monthly sex differences in unspecific modes were most pronounced during early preparation period (∼11 h in June).

### Periodization and performance characteristics

**FIGURE 4:**
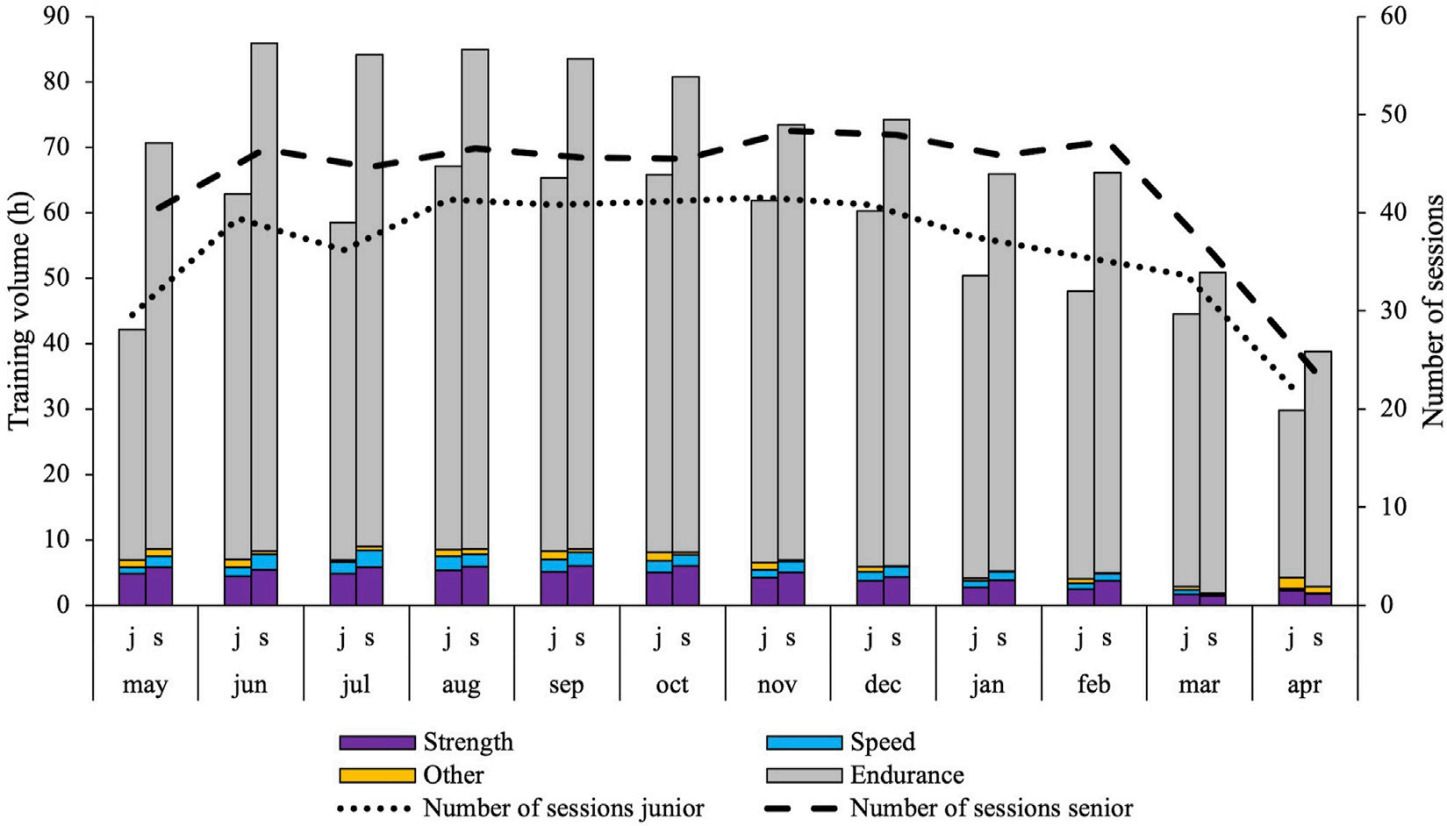
Monthly periodization of training forms (endurance, strength, speed, other) and number of training sessions in world-class XC-skiers during their best junior (j) and senior (s) season.

**FIGURE 5:**
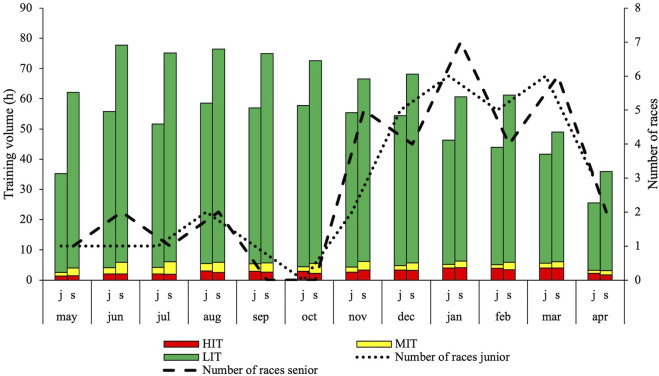
Monthly periodization of races and endurance training intensity distribution presented as total training in low-, moderate and high intensity in world-class XC-skiers during their best junior (j) and senior (s) season.

**FIGURE 6:**
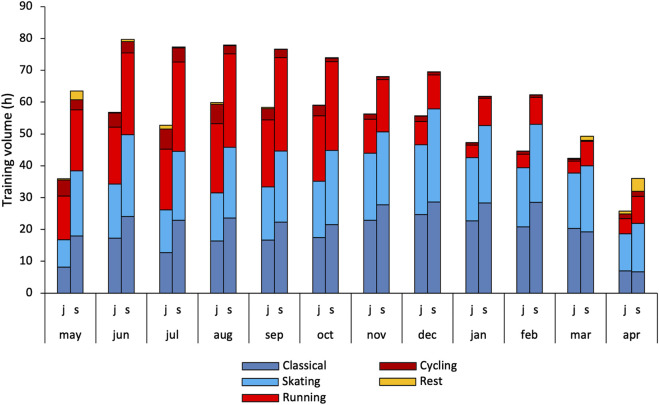
Monthly periodization of specific (skiing and roller skiing classical and skating) and unspecific (running, cycling and rest including rowing/kayak and other) modes in world-class XC-skiers during their best junior (j) and senior (s) season presented as total endurance and speed training.

Figure 4 shows the monthly distribution of training forms and the total number of sessions. The monthly TID expressed in absolute endurance training volume is shown in Figure 5 along with the number of races for each month. Figure 6 shows the distribution of training volume allocated on the different exercise modes.

### Development in aerobic power

Table 2 shows aerobic power during junior and senior age separated for women and men. The 3.8% change in absolute (L·min^−1^) VO_2max_ for women was not significant (*d* = 0.8) but relative VO_2max_ increased by 6.7% (*d* = 1.3, *p* = .012). Men increased VO_2max_ both in absolute (8.0%, *d* = 1.7, *p* = .009) and relative terms (5.6%, *d* = 1.4, *p* = .016). Sex differences in VO_2max_ were 29.5% and 35.0% at junior and senior age, respectively. When expressed relative to body weight, the corresponding sex differences were 15.5% and 14.4%, respectively.

**TABLE 2:** Highest reported aerobic power in world-class XC-skiers during junior age and best senior season.

Subject	Sex	Junior			Senior		
		VO_2max_ (L*min^−1^)	VO_2max_ (ml*kg^−1^*min^−1^)	Body mass (kg)	VO_2max_ (L*min^−1^)	VO_2max_ (ml*kg^−1^*min^−1^)	Body mass (kg)
1	F	4.97	69.4	71.6	4.89	69.3	70.6
2	F	4.46	64.9	68.7	4.71	74.3	63.4
3	F	3.74	64,2	58.3	4.04	69.7	58.0
4	F	4.44	63.9	69.5	4.58	69.4	66.0
5	F	4.40	64.0	68.8	4.34	68.8	63.1
6	F	3.79	74,8	50.7	4.25	80.0	53.1
7	F	4.46	66.3	67.3	4.39	67.4	65.1
Mean ± SD	F	4.31 ± 0.4	66.9 ± 4.0	64.6 ± 7.5	4.46 ± 0.3	71.3 ± 4.4*	62.8 ± 5.7
8	M	—	—	—	—	—	—
9	M	5.36	73.4	73.0	5.87	78.9	74.4
10	M	—	—	—	—	—	—
11	M	—	—	—	—	—	—
12	M	6.08	79.6	76.4	6.80	86.5	78.6
13	M	5.85	78.7	74.3	5.94	79.5	74.7
14	M	—	—	—	—	—	—
15	M	5.79	74.3	77.9	6.37	79.4	80.2
16	M	5.67	78.2	72.5	5.82	78.6	74.1
17	M	4,75	79.3	59.9	5.33	86.4	61.7
Mean ± SD	M	5.58 ± 0.5	77.3 ± 2.7	72.3 ± 6.4	6.02 ± 0.5*	81.6 ± 3.8*	73.9 ± 6.5**

VO_2max_, maximal oxygen uptake

* significant change from junior to senior age (*p* < 0.05) with large effect.

** significant change from junior to senior age (*p* < 0.05) with very large effect.

## Discussion

This is the first study to retrospectively compare training characteristics and aerobic power between the most successful junior and senior season in world-class XC-skiers. The main findings are as follows: 1) all skiers increased their total training volume from junior to senior age, with an average increase of approximately 200 h over an average span of 8.5 years; 2) junior training volume showed no association with senior training volume, but higher initial volumes were negatively correlated with increases in total volume; 3) the rise in total volume was primarily resulted from increased volume of LIT, complemented by increased MIT, while HIT remained unchanged which led to a shift from prevalent HIT to prevalent MIT in intensive training; 4) the sole sex differences in training characteristics were that women engaged in more strength training and unspecific modes during senior age, and they increased the extent of strength training and unspecific modes to a greater degree than men; 5) men increased both absolute and relative VO_2max_, whereas women only showed an increase in the latter.

### Total volume

World-class XC-skiers completed approximately 35% more annual training hours and ∼20% more sessions during the senior compared to the junior season, spanning an average of 8.5 years. The total training volume during the senior season was ∼860 h distributed across ∼520 sessions, consistent with previous reports on elite XC-skiers (Tønnessen et al., 2014; Sandbakk et al., 2016; Sandbakk and Holmberg, 2017; Solli et al., 2017). The total junior volume of ∼660 h discovered in this study is ∼25% higher than the volume during early senior age (21 years) reported for the most successful female XC-skier and male biathlete in history (Solli et al., 2017; Schmitt et al., 2021). Consequently, the observed 35% increase in training volume ranks at the lower end of previous case studies on long-term development from early twenties to peak age in winter endurance atheletes, which ranged from 27% to 80% (Solli et al., 2017; Rasdal et al., 2018; Schmitt et al., 2021; Solli et al., 2023). Given that no significant correlation emerged between junior training volume and generational aspects (year of birth) or junior age performance level (number of junior world championship medals) these factors can be ruled out as potential explanations. Thus, speculation remains as the only recourse, leaving room to consider individual training history as a contributory element.

This study establishes the first reference values for junior training volume in world-class XC skiers. It is intriguing to note that the total training volume at junior age did not correlate with senior training volume. However, athletes with higher training volume during junior age showed a smaller increase in training compared to those with lower junior training volume. Specifically, changes in training volume to senior age were approximately 1 h lower for every hour of higher training volume during junior age. This adds novel insights into the association between training volume at earlier and later career stages, suggesting that the increase in total volume is more tied to training history (such as junior training volume) than age in itself. This aligns with a broad range of individual percentage increases from junior to senior age, varying from 3% to 135%. Although previous case studies have reported a nonlinear change in total training volume with lower change rates as athletes approach age(s) of peak performance (Pinot and Grappe, 2014; Solli et al., 2017) there is a need for more research on the continuous development in training from junior to senior age (Staff et al., 2023). With both total volume and number of sessions increasing from junior to senior age, detailed investigations into design of sessions utilized by world-class athletes at both stages and any potential alterations could yield valuable supplementery information to the understanding of long-term training development.

Although men and women race different distances, no sex differences emerged in terms of total training volume or changes in the transition from junior to senior. However, sprint specialists increased their training to a lesser degree than distance athletes. This could be due to lower peak age among sprint athletes (Walther et al., 2022), suggesting that they are closer to their age of peak performance at late junior age, leading to a less pronounced rise in training volume. This is reinforced by the trend of younger ages during the analyszed senior season in sprint skiers in this study.

### Training forms

While endurance training increased by around 200 h (large effect), other training forms diminished by approximately 6 h (moderate effect) from junior to senior age, whereas strength and speed training remained unchanged. This underscores that the changes in total volume described above are almost solely driven by changes in endurance training. The senior season endurance volume of around 780 h (∼91% of total training) echoes previous studies (Tønnessen et al., 2014; Sandbakk and Holmberg, 2017; Solli et al., 2017). Notably, the junior endurance volume of ∼580 h was lower than reports by Karlsson et al. (2021) where junior skiers at lower performance level were studied. Despite this, no direct link between endurance training volume at junior age and performance level is indicated and more investigations on the relationship between performance level and training volume across age groups are needed to fully understand this interaction in endurance sports. The proportion of strength and speed training in this study was 9.5% and 8.3% during junior and senior age, respectively, aligning with previous research on senior XC-skiers (Tønnessen et al., 2014; Sandbakk and Holmberg, 2017; Solli et al., 2017). Interestingly, we observed large sex differences in strength training at senior age but not at junior age. Women increased their strength training volume while men showed a reduction on corresponding volume, resulting in considerable sex differences in the transition from junior to senior age. Strength training can positively affect XC performance by enhancing work economy (Stöggl and Holmberg, 2022). The shorter distances in women’s races may put relatively higher demands on power output on women. Similarly, sex-differences in XC-skiing are influenced by the contribution of poling (Sandbakk et al., 2014), where the upper body is strongly involved in propulsion. Women may therefore rely on strength training to diminish this gap. However, intervention studies of heavy strength training effects on double poling performance in female XC-skiers have shown contradicting outcomes (Skattebo et al., 2014; Østerås et al., 2016). Consequently other aspects might be important for the sex differences in strength training found in the present study. Thus, Jones et al. (2021b) reported that increased body mass, increased upper body lean mass and decreased whole body fat mass were good predictors of performance changes in female XC-skiers, but not in males. These parameters are potentially influenced by strength training and it seems plausible that women use such training to compensate for generaly lower muscle mass compared to men which is specially pronounced in the upper body. We assume that strength training thereby contributes to maintain or even increase muscle mass while performing large amounts of endurance training. Future studies on strength training in XC-sking should therefore consider both sex differences and body composition.

Previous studies have reported that sprint skiers implement more strength and speed training than distance skiers (Losnegard and Hallén, 2014; Sandbakk and Holmberg, 2017). However, this was not observed in the present participants. One plausible reason could be that the present athletes aimed to be more versatile and succeed in both sprint and distance races. Another reason could be that race characteristics in sprint races have changed over the last decade, with slightly longer distances, more demanding track profiles and fewer flat races arranged in city centers.

### Endurance training

Nearly all changes in training volume from junior to senior age could be explained by the 40% increase in LIT. While MIT increased modestly in absolute terms, the relative mean change was as high as 81%. In contrast, the volume of HIT remained unchanged. Increases in LIT over time have been observed in previous studies of XC-skiing (Solli et al., 2017) and other endurance sports (Tjelta, 2013; Schmitt et al., 2021). The most successful female XC-skier and a world-class biathlete both showed similar trends in changes of MIT and HIT (Solli et al., 2017; Solli et al., 2023), while reports from other sports vary (Staff et al., 2023). In addition to the high proportion of LIT, the present athletes performed more HIT than MIT at junior age. HIT has been shown to have a greater short-term effect on endurance performance and physiological adaptations (e.g., increased VO_2max_) than LIT and MIT (Laursen and Jenkins, 2002; Stöggl and Sperlich, 2014). These changes are related to superior cardiovascular characteristics such as increased stroke volume, possibly explaining relatively more HIT at junior age where this development is particularly important (Helgerud et al., 2007; Bjerring et al., 2019). However, the high stress induced by HIT may increase the odds of performance stagnation and overtraining if used too frequently (Billat et al., 1999), while the lower stress of LIT and MIT allows for high training volume and frequency.

A longitudinal increase in training load is likely necessary to achieve long-term success in endurance sports (Jones, 2006; Solli et al., 2017; Haugen et al., 2022). Interestingly, the athletes in this study achieved this change by increasing LIT and MIT, while HIT remained unchanged. Even though the tolerable total training stress is elevated by adaptations to training over time, it is still limited, and an athlete cannot increase both LIT, MIT and HIT equally. One could hypothesize that there is an individual maximum of tolerable HIT that is closely related to total training volume, and athletes may have to choose between further increases in HIT or total training volume. This hypothesis is partly supported by Solli et al. (2019) who reported that a period of increased HIT at senior age was associated with a lower total volume of endurance training compared to a different period with a higher volume of training where HIT was downregulated. Overall, there are several possible explanations for why the present athletes chose to increase LIT and MIT (and thereby total training volume) and not HIT in their senior ages. First, performance factors whose development is particularly promoted by HIT, such as VO_2max_, are already developed to a very high level by the end of junior age. Second, more LIT and MIT enable athletes to train more frequently and with a higher volume, promoting the development of other key performance factors such as work economy and speed at the lactate threshold (Jones, 2006). Third, athletes are already at a high level and can perform LIT and MIT at higher speeds, making this training more relevant from a technical perspective. This also allows them to train at a high percentage of VO_2max_ at submaximal intensities. Lastly, there is evidence suggesting that key regulators of exercise-induced mitochondrial biogenesis, which is crucial for adaptations to endurance training, are diminished by high volumes of HIT (Granata et al., 2020).

Interestingly, the most pronounced changes in LIT from junior to senior age occurred during early preparation. We hypothesize that this is a consequence of natural progression and tolerance for higher training loads, as well as a higher degree of professionalization at senior age which is supported by a recent case study in biathlon (Solli et al., 2023). A high volume of training must be achieved throughout the year while ensuring that stress induced by training is at a sufficiently low level during competition season. While HIT outweighs MIT for most of the annual training year at junior age, the increased volume of LIT and MIT during preparation leads to a split of the season in TID characteristics at senior age. Here, MIT clearly outweighs HIT from the start of the training season, but this ratio is reversed as the competition season approaches. This intensification has been described previously in XC-skiing (Solli et al., 2017; Talsnes et al., 2023). Although TID and especially portions of intensive training are highly debated (Burnley et al., 2022; Foster et al., 2022), there is some evidence suggesting that an increased percentage of HIT and a shift from a predominance of MIT to a predominance of HIT, as observed during the senior season in this study, is beneficial for endurance performance (Sylta et al., 2017; Filipas et al., 2021). However, it must be mentioned that the observed changes both from junior to senior and within the senior season are rather small, as 91% and 92% of the endurance training is performed as LIT during junior and senior age, respectively. Future research should therefore focus on the analysis of LIT to better understand the specific effects of such training. Furthermore, analyses on how training is distributed across different types of endurance sessions should be conducted. This would add complementary value to the understanding of TID applied by world-class skiers.

Both specific and unspecific modes increased from junior to senior age, but the distribution remained unchanged on an annual level. The present proportions of specific and unspecific modes are in line with previous reports of XC-skiers (Tønnessen et al., 2014; Solli et al., 2017; Talsnes et al., 2023). While specific and unspecific modes were equally distributed during the preparation period, mainly specific modes were undertaken during the competition period at both the junior and senior age. Such cross training is a commonly used strategy to tolerate high training volumes while avoiding injuries and overload (Sandbakk et al., 2021). Reduced training volume towards the competition season allows for a more unilateral application of training modes. This shift is somewhat less distinct during senior age, as a slightly higher proportion of specific modes during summer and a lower proportion during winter were observed. This can be explained by the generally higher training volume at senior age, making the organization and variation in modes even more critical. Interestingly, women applied a higher volume and a higher proportion of unspecific modes at senior age than men, and this difference was most pronounced during the early preparation season. Our data suggest two possible explanations for this. First, the observed sex differences in strength training put higher demands on the upper body making cross-training with unspecific movements primarily involving the lower body a favorable choice. Second, six of the seven women applied altitude training camps during summer, while only five of ten men attended such camps. Summer altitude camps are typically conducted in places associated with poorer conditions for roller-skiing, and running becomes a more favored option.

### Aerobic power

This study pioneers a retrospective analysis of aerobic power development in world-class XC-skiers from junior to senior age. While the investigated men increased their absolute VO_2max_ by 8%, no mean change was seen among women. Hence, sex differences in absolute VO_2max_ were more pronounced at senior age. Given that XC-skiing is weight-bearing, VO_2max_ relative to body mass is crucial and a good predictor of performance. In this aspect, men and women both exhibited 6%–7% increase in relative VO_2max_. The reasons for the divergent changes of absolute and relative VO_2max_ for men and women are unknown. One possible explanation could be changes in women’s body composition with increased fat mass during adolescence, later reduced by high training volumes and leading to higher relative VO_2max_. The slower speed of women compared to men may result in more uphill time (Solli et al., 2020). As relative VO_2max_ is more important in uphills compared to flat sections, women may have to maximize their relative VO_2max_ to a larger degree than men. This can partly be achieved by a reduction in body mass. This tendency was also observed in this study, although body mass only changed significantly in men.

Remarkably, junior VO_2max_ values found in this study are surpass prior reports on tier 4 junior skiers (Sandbakk et al., 2011; Zoppirolli et al., 2020). The present junior VO_2max_ values even outstrip those reported among national level senior skiers (Sandbakk et al., 2010) and the present senior values are among the highest ever reported for endurance athletes (Tønnessen et al., 2014; Haugen et al., 2018b).

Regression analyses between aerobic power changes and selected training parameters were not significant. Yet, it is plausible that the observed changes in LIT and MIT promote submaximal factors such as speed and fractional utilization of VO_2max_ at lactate threshold or work economy (Jones, 2006). These factors likely facilitate senior age performance development (Staff et al., 2023). Therefore, future studies should concurrently examine long-term development in VO_2max_, submaximal responses, training characteristics, and direct performance measurement.

### Strengths and limitations

This study has multiple strengths and limitations. Unlike typical case reports on elite athletes, this study provides extensive insights by studying 17 medal-winning athletes simultaneously. Including both men and women enhances the findings’ applicability. To limit generational influences, athletes born between 1972 and 1998 were included. With no correlations between year of birth and training characteristics, the effect of time-specific training philosophy is minimized. However, recall bias is a potential error source especially among older participants, and training might be influenced by historical coaching philosophies. Finally, the study might not account for changes in race calendars and traveling routines for six athletes who had their best senior season during the COVID-19 pandemic.

### Practical application

This study’s insights into the training characteristics and aerobic power development among world-class XC-skiers from junior to senior age provide invaluable understanding for long-term athlete development. This framework can guide coaches and sports federations in planning and evaluating in future XC skiers’ developmental trajectories.

Our data indicates that world-class XC-skiers have developed a very high peak aerobic power already at junior age, while the further improvements in aerobic power and endurance performance seem to be driven by an increase in LIT and MIT. However, this increase in training volume was less prominent for athletes with initially high junior training volumes, suggesting that individual training background should be considered when planning for an increase in training volume.

Apart from women applying more training in unspecific modes at senior age, no sex differences were found in long-term endurance training. Still, other characteristics such as session design, speed, or the choice of terrain and friction in roller-skis might vary between men and women. Women increased their strength training to a greater degree and applied more strength training at senior age than men who showed a decrease from junior to senior age. It could be speculated that strength training is particularly important for women to retain muscle mass, especially in the upper body, while concurrently applying high volumes of endurance training.

Sprint and distance skiers showed similar long-term training characteristics, except for a higher increase in total training volume from junior to senior age in distance skiers. Overall, this suggests that sprint and distance races require relatively similar training patterns.

## Conclusion

This study establishes the first reference values of training characteristics and aerobic power in the best junior and senior seasons of medal-winning, world-class XC-skiers. The change in training was characterized by an increase of ∼200 h, primarily achieved through increased LIT (∼185 h) supplemented by a small increase in MIT (∼13 h). This highlights the importance of LIT and MIT for the long-term development of endurance performance, intensities that are underrepresented in the scientific literature. Training volume at junior age was not related to volume at senior age but was negatively associated with the increase in total volume from junior to senior age. This underscores the relevance of individual training background when analyzing and planning the further development of training characteristics. In addition to increased LIT, both men and women shifted from a prevalence of HIT towards a prevalence of MIT in their TID, indicating that achieving a world-class endurance level requires higher training volume and altered TID throughout the career. Divergent trends in strength training were observed between sexes, suggesting a greater need for developing and maintaining strength and body composition in women. Our findings of exceptionally high VO_2max_ at junior age further increased by 8% in men but not in women, and the increase of 6%–7% in relative VO_2max_ in both sexes confirms the importance of a high VO_2max_ to achieve a world-class endurance level and highlight the relevance of long-term development.

## Data Availability

The raw data supporting the conclusions of this article will be made available by the authors, without undue reservation.

## References

[B1] Allen S. V. Hopkins W. G. (2015). Age of peak competitive performance of elite athletes: a systematic review. Sports Med. 45, 1431–1441. 10.1007/s40279-015-0354-3 26088954

[B2] Bell P. G. Furber M. J. W. Someren K. A. van Antón-Solanas A. Swart J. (2017). The Physiological Profile of a Multiple Tour de France Winning Cyclist. Med. Sci. Sports Exerc 49, 115–123. 10.1249/mss.0000000000001068 27508883

[B3] Billat V. L. Flechet B. Petit B. Muriaux G. Koralsztein J. P. (1999). Interval training at V˙O2max: effects on aerobic performance and overtraining markers. Med. Sci. Sports Exerc 31, 156–163. 10.1097/00005768-199901000-00024 9927024

[B4] Bjerring A. W. Landgraff H. E. Stokke T. M. Murbræch K. Leirstein S. Aaeng A. (2019). The developing athlete’s heart: a cohort study in young athletes transitioning through adolescence. Eur. J. Prev. Cardiol. 26, 2001–2008. 10.1177/2047487319862061 31284749

[B5] Burnley M. Bearden S. E. Jones A. M. (2022). Polarized training is not optimal for endurance athletes. Med. Sci. Sports Exerc. 54, 1032–1034. 10.1249/mss.0000000000002869 35135998

[B6] Edvardsen E. Hem E. Anderssen S. A. (2014). End criteria for reaching maximal oxygen uptake must Be strict and adjusted to sex and age: a cross-sectional study. PLoS ONE 9, e85276. 10.1371/journal.pone.0085276 24454832 PMC3891752

[B7] Erp T. van Sanders D. Koning J. J. de (2020). Training characteristics of male and female professional road cyclists: a 4-year retrospective analysis. Int. J. Sport Physiol. 15, 534–540. 10.1123/ijspp.2019-0320 31722298

[B8] Filipas L. Bonato M. Gallo G. Codella R. (2021). Effects of 16 weeks of pyramidal and polarized training intensity distributions in well‐trained endurance runners. Scand. J. Med. Sci. Spor 32, 498–511. 10.1111/sms.14101 PMC9299127 34792817

[B9] Foster C. Casado A. Esteve-Lanao J. Haugen T. Seiler S. (2022). Polarized training is optimal for endurance athletes. Med. Sci. Sports Exerc. 54, 1028–1031. 10.1249/mss.0000000000002871 35136001

[B10] Granata C. Oliveira R. S. F. Little J. P. Bishop D. J. (2020). Forty high-intensity interval training sessions blunt exercise-induced changes in the nuclear protein content of PGC-1α and p53 in human skeletal muscle. Am. J. Physiol.-Endocrinol. Metab. 318, E224–E236. 10.1152/ajpendo.00233.2019 31794264 PMC7052577

[B11] Haugen T. A. Solberg P. A. Foster C. Morán-Navarro R. Breitschädel F. Hopkins W. G. (2018a). Peak age and performance progression in world-class track-and-field athletes. Int. J. Sport Physiol. 13, 1122–1129. 10.1123/ijspp.2017-0682 29543080

[B12] Haugen T. Paulsen G. Seiler S. Sandbakk Ø. (2018b). New records in human power. Int. J. Sport Physiol. 13, 678–686. 10.1123/ijspp.2017-0441 28872385

[B13] Haugen T. Sandbakk Ø. Seiler S. Tønnessen E. (2022). The training characteristics of world-class distance runners: an integration of scientific literature and results-proven practice. Sports Med. - Open 8, 46. 10.1186/s40798-022-00438-7 35362850 PMC8975965

[B14] Helgerud J. Høydal K. Wang E. Karlsen T. Berg P. Bjerkaas M. (2007). Aerobic high-intensity intervals improve V˙O2max more than moderate training. Med. Sci. Sports Exerc 39, 665–671. 10.1249/mss.0b013e3180304570 17414804

[B15] Hopkins W. G. Marshall W. S. Batterham A. M. Hanin J. (2009). Progressive statistics for studies in sports medicine and exercise science. Med. Sci. Sports Exerc 41, 3–13. 10.1249/mss.0b013e31818cb278 19092709

[B16] Ingjer F. (1991). Maximal oxygen uptake as a predictor of performance ability in women and men elite cross‐country skiers. Scand. J. Med. Sci. Spor 1, 25–30. 10.1111/j.1600-0838.1991.tb00267.x

[B17] International Ski Federation (2023). International ski federation. Available at: https://www.fis-ski.com/en/inside-fis/document-library/cross-country-documents (Accessed June 5, 2023).

[B18] Jones A. M. Kirby B. S. Clark I. E. Rice H. M. Fulkerson E. Wylie L. J. (2021a). Physiological demands of running at 2-hour marathon race pace. J. Appl. Physiol. 130, 369–379. 10.1152/japplphysiol.00647.2020 33151776

[B19] Jones T. W. Lindblom H. P. Karlsson Ø. Andersson E. P. McGawley K. (2021b). Anthropometric, physiological, and performance developments in cross-country skiers. Med. Sci. Sports Exerc 53, 2553–2564. 10.1249/mss.0000000000002739 34649265

[B20] Jones A. M. (2006). The physiology of the world record holder for the women’s marathon. Int. J. Sports Sci. Coa 1, 101–116. 10.1260/174795406777641258

[B21] Joyner M. J. Coyle E. F. (2008). Endurance exercise performance: the physiology of champions. J. Physiol. 586, 35–44. 10.1113/jphysiol.2007.143834 17901124 PMC2375555

[B22] Karlsson Ø. Laaksonen M. S. McGawley K. (2021). Training and illness characteristics of cross-country skiers transitioning from junior to senior level. Plos One 16, e0250088. 10.1371/journal.pone.0250088 33989314 PMC8121355

[B23] Laursen P. B. Jenkins D. G. (2002). The scientific basis for high-intensity interval training: optimising training programmes and maximising performance in highly trained endurance athletes. Sports Med. 32, 53–73. 10.2165/00007256-200232010-00003 11772161

[B24] Losnegard T. Hallén J. (2014). Physiological differences between sprint- and distance-specialized cross-country skiers. Int. J. Sport Physiol. 9, 25–31. 10.1123/ijspp.2013-0066 24155024

[B25] Losnegard T. Myklebust H. Spencer M. Hallén J. (2013). Seasonal variations in VO2max, O2-cost, O2-deficit, and performance in elite cross-country skiers. J. Strength Cond. Res. 27, 1780–1790. 10.1519/jsc.0b013e31827368f6 22996025

[B26] McKay A. K. A. Stellingwerff T. Smith E. S. Martin D. T. Mujika I. Goosey-Tolfrey V. L. (2022). Defining training and performance caliber: a participant classification framework. Int. J. Sport Physiol. 17, 317–331. 10.1123/ijspp.2021-0451 34965513

[B27] Midgley A. W. McNaughton L. R. Polman R. Marchant D. (2007). Criteria for determination of maximal oxygen uptake: a brief critique and recommendations for future research. Sports Med. 37, 1019–1028. 10.2165/00007256-200737120-00002 18027991

[B28] Østerås S. Welde B. Danielsen J. Tillaar R. van den Ettema G. Sandbakk Ø. (2016). Contribution of upper-body strength, body composition, and maximal oxygen uptake to predict double poling power and overall performance in female cross-country skiers. J. Strength Cond. Res. 30, 2557–2564. 10.1519/jsc.0000000000001345 26817743

[B29] Pinot J. Grappe F. (2014). A six-year monitoring case study of a top-10 cycling Grand Tour finisher. J. Sport Sci. 33, 907–914. 10.1080/02640414.2014.969296 25357188

[B30] Rasdal V. Moen F. Sandbakk Ø. (2018). The long-term development of training, technical, and physiological characteristics of an olympic champion in nordic combined. Front. Physiol. 9, 931. 10.3389/fphys.2018.00931 30061843 PMC6055063

[B31] Sandbakk Ø. Holmberg H.-C. (2017). Physiological capacity and training routines of elite cross-country skiers: approaching the upper limits of human endurance. Int. J. Sport Physiol. 12, 1003–1011. 10.1123/ijspp.2016-0749 28095083

[B32] Sandbakk Ø. Holmberg H.-C. Leirdal S. Ettema G. (2010). Metabolic rate and gross efficiency at high work rates in world class and national level sprint skiers. Eur. J. Appl. Physiol. 109, 473–481. 10.1007/s00421-010-1372-3 20151149

[B33] Sandbakk Ø. Welde B. Holmberg H.-C. (2011). Endurance training and sprint performance in elite junior cross-country skiers. J. Strength Cond. Res. 25, 1299–1305. 10.1519/jsc.0b013e3181d82d11 21081854

[B34] Sandbakk Ø. Ettema G. Holmberg H.-C. (2014). Gender differences in endurance performance by elite cross‐country skiers are influenced by the contribution from poling. Scand. J. Med. Sci. Spor 24, 28–33. 10.1111/j.1600-0838.2012.01482.x 22621157

[B35] Sandbakk Ø. Hegge M. Losnegard T. Skattebo Ø. Tønnessen E. Holmberg H.-C. (2016). The physiological capacity of the world’s highest ranked female cross-country skiers. Med. Sci. Sports Exerc 48, 1091–1100. 10.1249/mss.0000000000000862 26741124 PMC5642331

[B36] Sandbakk Ø. Haugen T. Ettema G. (2021). The influence of exercise modality on training load management. Int. J. Sport Physiol. 16, 605–608. 10.1123/ijspp.2021-0022 33639611

[B37] Schmitt L. Bouthiaux S. Millet G. P. (2021). Eleven years’ monitoring of the world’s most successful male biathlete of the last decade. Int. J. Sport Physiol. 16, 900–905. 10.1123/ijspp.2020-0148 32887848

[B38] Seiler K. S. Kjerland G. Ø. (2006). Quantifying training intensity distribution in elite endurance athletes: is there evidence for an “optimal” distribution? Scand. J. Med. Sci. Spor 16, 49–56. 10.1111/j.1600-0838.2004.00418.x 16430681

[B39] Skattebo Ø. Hallén J. Rønnestad B. R. Losnegard T. (2014). Maximal strength training does not improve double polingperformance in well trained junior female cross country skiers. Ann. Res. Sport Phys. Act., 45–46. 10.14195/2182-7087_5_8

[B40] Solli G. S. Tønnessen E. Sandbakk Ø. (2017). The training characteristics of the world’s most successful female cross-country skier. Front. Physiol. 8, 1069. 10.3389/fphys.2017.01069 29326603 PMC5741652

[B41] Solli G. S. Tønnessen E. Sandbakk Ø. (2019). Block vs. Traditional periodization of HIT: two different paths to success for the world’s best cross-country skier. Front. Physiol. 10, 375. 10.3389/fphys.2019.00375 31024338 PMC6460991

[B42] Solli G. S. Kocbach J. Sandbakk S. B. Haugnes P. Losnegard T. Sandbakk Ø. (2020). Sex-based differences in sub-technique selection during an international classical cross-country skiing competition. PLoS ONE 15, e0239862. 10.1371/journal.pone.0239862 32991633 PMC7523995

[B43] Solli G. S. Flom A. H. Talsnes R. K. (2023). Long-term development of performance, physiological, and training characteristics in a world-class female biathlete. Front. Sports Act. Living 5, 1197793. 10.3389/fspor.2023.1197793 37398554 PMC10308379

[B44] Sollie O. Losnegard T. (2022). Sex differences in physiological determinants of performance in elite adolescent, junior, and senior cross-country skiers. Int. J. Sports Physiol. Perform. 17, 1304–1311. 10.1123/ijspp.2021-0366 35894954

[B45] Staff H. C. Solli G. S. Osborne J. O. Sandbakk Ø. (2023). Long-term development of training characteristics and performance-determining factors in elite/international and world-class endurance athletes: a scoping review. Sports Med. 53, 1595–1607. 10.1007/s40279-023-01850-z 37178349 PMC10356634

[B46] Stöggl T. Holmberg H.-C. (2022). A systematic review of the effects of strength and power training on performance in cross-country skiers. J. Sport Sci. Med. 21, 555–579. 10.52082/jssm.2022.555 PMC9741725 36523891

[B47] Stöggl T. Sperlich B. (2014). Polarized training has greater impact on key endurance variables than threshold, high intensity, or high volume training. Front. Physiol. 5, 33. 10.3389/fphys.2014.00033 24550842 PMC3912323

[B48] Sylta Ø. Tønnessen E. Seiler S. (2014a). Do elite endurance athletes report their training accurately? Int. J. Sport Physiol. 9, 85–92. 10.1123/ijspp.2013-0203 23921186

[B49] Sylta Ø. Tønnessen E. Seiler S. (2014b). From heart-rate data to training quantification: a comparison of 3 methods of training-intensity analysis. Int. J. Sport Physiol. 9, 100–107. 10.1123/ijspp.2013-0298 24408353

[B50] Sylta Ø. Tønnessen E. Sandbakk Ø. Hammarström D. Danielsen J. Skovereng K. (2017). Effects of high-intensity training on physiological and hormonal adaptions in well-trained cyclists. Med. Sci. Sports Exerc 49, 1137–1146. 10.1249/mss.0000000000001214 28121800

[B51] Talsnes R. K. Moxnes E. F. Nystad T. Sandbakk Ø. (2023). The return from underperformance to sustainable world-class level: a case study of a male cross-country skier. Front. Physiol. 13, 1089867. 10.3389/fphys.2022.1089867 36699686 PMC9870290

[B52] Tjelta L. I. (2013). A longitudinal case study of the training of the 2012 European 1500m track champion. Ijass Int. J. Appl. Sports Sci. 25, 11–18. 10.24985/ijass.2013.25.1.11

[B53] Tønnessen E. Sylta Ø. Haugen T. A. Hem E. Svendsen I. S. Seiler S. (2014). The road to gold: training and peaking characteristics in the year prior to a gold medal endurance performance. Plos One 9, e101796. 10.1371/journal.pone.0101796 25019608 PMC4096917

[B54] Tønnessen E. Haugen T. A. Hem E. Leirstein S. Seiler S. (2015). Maximal aerobic capacity in the winter-olympics endurance disciplines: olympic-medal benchmarks for the time period 1990–2013. Int. J. Sport Physiol. 10, 835–839. 10.1123/ijspp.2014-0431 25611016

[B55] Walther J. Mulder R. Noordhof D. A. Haugen T. A. Sandbakk Ø. (2022). Peak age and relative performance progression in international cross-country skiers. Int. J. Sport Physiol. 17, 31–36. 10.1123/ijspp.2021-0065 34186511

[B56] Zoppirolli C. Modena R. Fornasiero A. Bortolan L. Skafidas S. Savoldelli A. (2020). Talent development in young cross-country skiers: longitudinal analysis of anthropometric and physiological characteristics. Front. Sports Act. Living 2, 111. 10.3389/fspor.2020.00111 33345100 PMC7739632

